# A simulation of diffractive liquid crystal smart window for privacy application

**DOI:** 10.1038/s41598-022-15636-2

**Published:** 2022-07-05

**Authors:** Chan-Hee Han, Hyeonseok Eo, Tae-Hoon Choi, Wook-Sung Kim, Seung-Won Oh

**Affiliations:** 1 Department of Electrical Information Communication Engineering, Kangwon National University, Samcheok, Gangwon 25913 Republic of Korea; 2 Department of Electrical Engineering, POSTECH, Pohang, 37673 Republic of Korea; 3 Smart Materials R&D Center, Korea Automotive Technology Institute, Cheonan, 31214 Republic of Korea

**Keywords:** Liquid crystals, Displays

## Abstract

Using a single substrate, we demonstrate a simple two-dimensional (2-D) phase grating cell with an octothorp electrode. Owing to the large spatial phase difference in any direction, the proposed grating cell has a high haze value in the opaque state (76.7%); Moreover, it has the advantages of a one-dimensional (1-D) phase grating cell, such as high fabricability, fast response time, and low operating voltage. Furthermore, the proposed grating cell has a faster response time than the 2-D grating cell (comparable to a 1-D grating cell). All the electro-optic parameters have been calculated using a commercial modeling tool. Consequently, we expect our proposed grating cell to find applications in virtual reality (VR)/augmented reality (AR) systems or window displays with fast response times.

## Introduction

Smart windows have been reported to control the transmittance of sunlight and solar heat in electrochromic, photochromic, thermochromic, suspended particle, and liquid crystal (LC) devices^1–10^. LC devices particularly benefit from a fast response time and the ability to adjust light scattering, absorption, or reflection, whereas other smart windows can only control light absorption^11–25^. LC windows can be utilized in privacy applications, augmented reality (AR), virtual reality (VR), and transparent displays by controlling light scattering^26–28^. Polymer structures, chiral dopants, and ions in LCs can be used to induce light scattering. However, these devices have some limitations including high operating voltage, slow response time, and lack of reliability^23,29^.

To overcome these drawbacks, LC grating devices have been developed for smart windows^30–35^. Although light diffraction using an LC phase grating is not the same as light scattering, it has the same impact on haze control. They have various benefits in terms of haze control, including reduced haze and a broad viewing angle in transparent conditions, low operating voltage, and fast response time. However, owing to the low haze value of 51%, they are not extensively used in one-dimensional (1-D) applications^31,32^. To overcome this drawback, two-dimensional (2-D) LC phase grating devices, consisting of top and bottom substrates with crossed interdigitated electrodes, have been proposed^33–35^. They had a haze value of 83.8%, which is quite high. On the other hand, the 2-D grating cells, have serious drawbacks, including a slow turn-off response time, a high operating voltage, and problematic manufacture owing to the difficulty of perpendicularly matching the top and bottom interdigitated electrodes in practice.

In this study, we demonstrate a simple 2-D LC phase grating cell with an octothorp electrode on a single substrate. The proposed grating cell has a high haze value in the opaque state (76.7%) because of a substantial spatial phase difference independent of the azimuthal angle, while also having 1-D grating cell advantages, such as easy fabrication, fast response time, and low operating voltage. The proposed grating cell can be used in VR/AR systems or window displays that require to have a fast response.

## Operating principle

**Figure 1:**
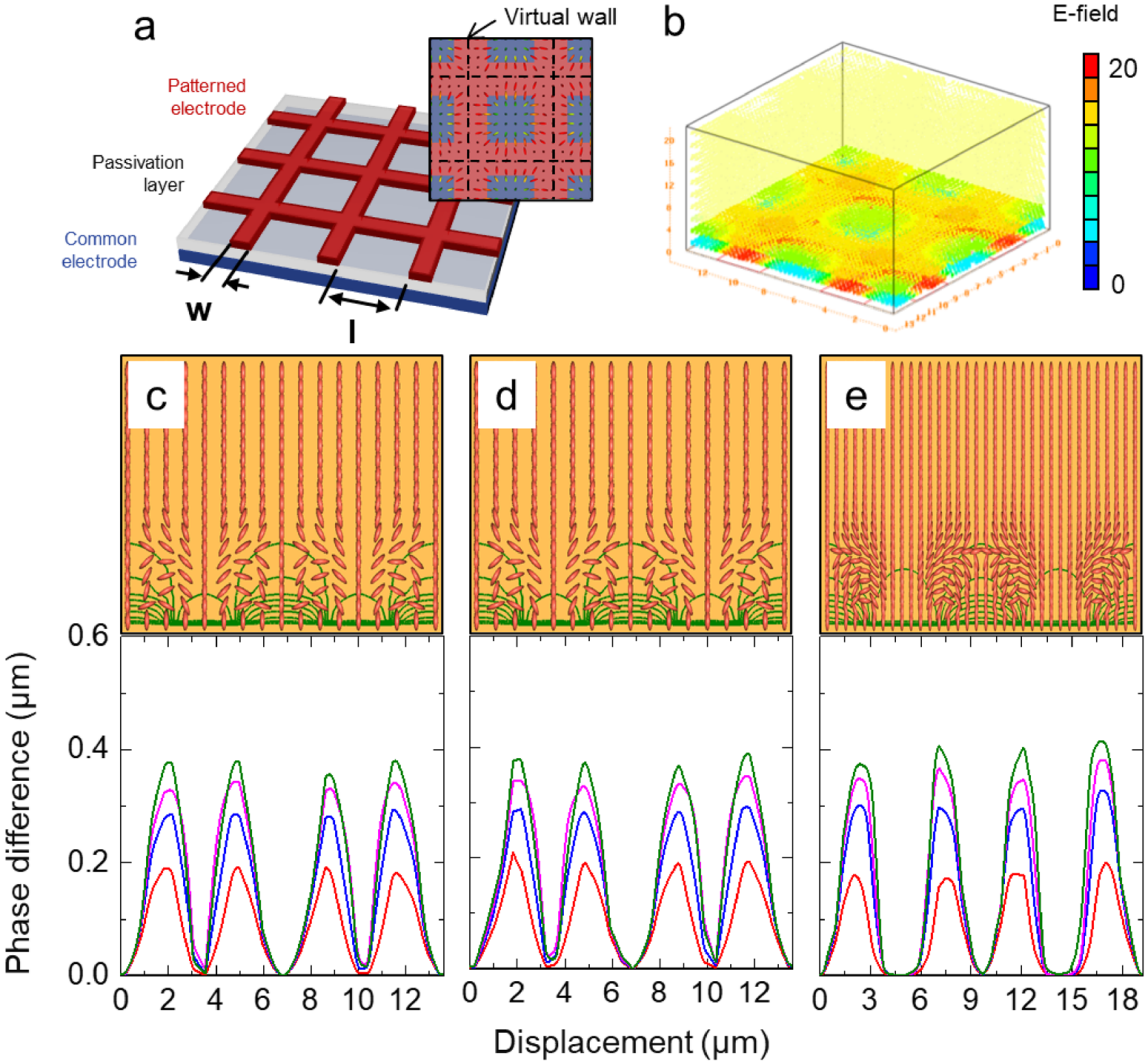
Proposed LC grating cell. (**a**) Cell structure and top-view of LC director configurations. (**b**) E-field distribution (20 V). Calculated LC director distributions and phase difference profiles in (**c**) *x*-direction, (**d**) *y*-direction, and (**e**) diagonal direction.

We estimated the electro-optical characteristics of the LC grating cell using the commercial modeling program TechWiz LCD 3D (Sanayi System Co., Ltd., Korea). A common electrode, passivation layer, and patterned electrode on the bottom substrate are shown in Fig. 1a as a representation of the proposed grating cell. The vertical and horizontal tracks of the octothorp are interconnected. The initial vertically aligned LC molecules are tilted down along the electric field directions using a patterned octothorp electrode (Fig. 1b), resulting in a substantial spatial phase difference along the vertical and horizontal directions. Furthermore, owing to the diffraction effect generated by the significant spatial phase difference, the proposed grating cell could be switched to an opaque state. The dotted black lines indicate the virtual wall where the LCs do not orient and act as a polymer wall (Fig. 1a).

Figure 1c,d, and e show the calculated LC director distributions and phase difference profiles in vertical, horizontal, and diagonal directions, respectively, while applying an electric field (15 V). Because the octothorp electrode is produced on the bottom substrate, the proposed LC grating cell exhibits a substantial spatial phase difference in the vertical, horizontal, and diagonal directions. Because more LC molecules are reoriented along the direction of the applied electric field owing to the octothorp electrode, the suggested grating cell shows the same spatial phase difference along the diagonal direction as it does along the vertical or horizontal direction, as shown in Fig. 1c–e. When an electric field is provided to the LC cell in this grating cell, a substantial spatial phase difference is created independent of the azimuth angle. As a result, when white light enters the LC cell, it is diffracted, allowing the LC cell to be switched to a suitable opaque state owing to a substantial spatial phase difference, regardless of azimuth angle.

## Results and discussion

To realize the goal of this study, the proposed grating cell possesses the characteristics of vertical alignment, positive nematic LC (such as E7, Merck) (dielectric anisotropy *Δε* = 13.8, refractive indices *n*_*o*_ = 1.52 and *n*_*e*_ = 1.75, elastic constants *k*_*11*_, *k*_*22*_, and *k*_*33*_ are 10.3, 7.4, and 16.5 pN, respectively), and octothorp electrode on the bottom substrate. The width, length, and cell gap of the patterned electrode were 2.8, 4, and 20 µm, respectively. In addition, we set the TechWiz LCD 3D options, such as a pretilt angle, azimuthal angle, and wavelength as 90°, 0°, and 543.5 nm, respectively; moreover, we used an optical analysis method with a 2 × 2 extended Jones matrix. The far-field intensity was detected using a photodiode located 30 cm away from the LC cell.

**Figure 2:**
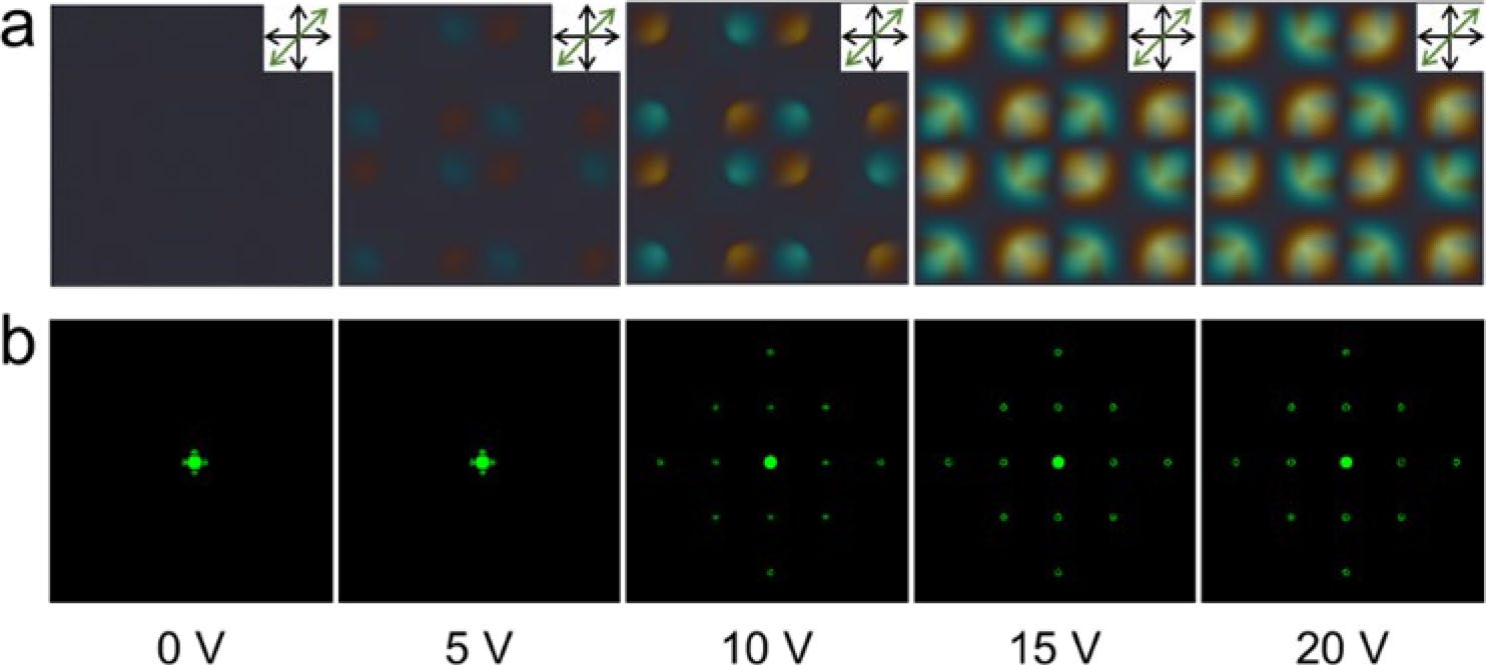
(**a**) POM images of the proposed grating with crossed polarizers and full-wave plate. (**b**) Diffraction pattern of the proposed grating at different applied voltages from 0 to 20 V.

Figure 2a shows the POM images of the proposed grating cell with crossed polarizers at various applied voltages. To verify the direction of rotation of the LCs, a full-wave plate (45°) was inserted between the crossed polarizers. When the voltage was increased, the brightness (retardation) of most regions increased, whereas the virtual wall's brightness (retardation) remained constant, resulting in a spatial phase difference^30–35^. Because of the spontaneous fluctuation of the phase difference, the created defect patterns worked well as 2D diffraction gratings^36^. Green diffraction patterns were detected on a dark screen when an unpolarized laser beam (543.5 nm) passed through the LC cell (Fig. 2b). Because most of the laser energy is directed to higher orders, the intensity of the zeroth-order is significantly reduced, regardless of the direction of polarization. We can observe that the diffraction energy is well transported from the zeroth-order to higher orders, regardless of the direction of polarization. Because of the significant spatial phase difference, we can expect the proposed grating cell with an octothorp electrode to switch to an excellent opaque state, regardless of the azimuth angle.

**Figure 3:**
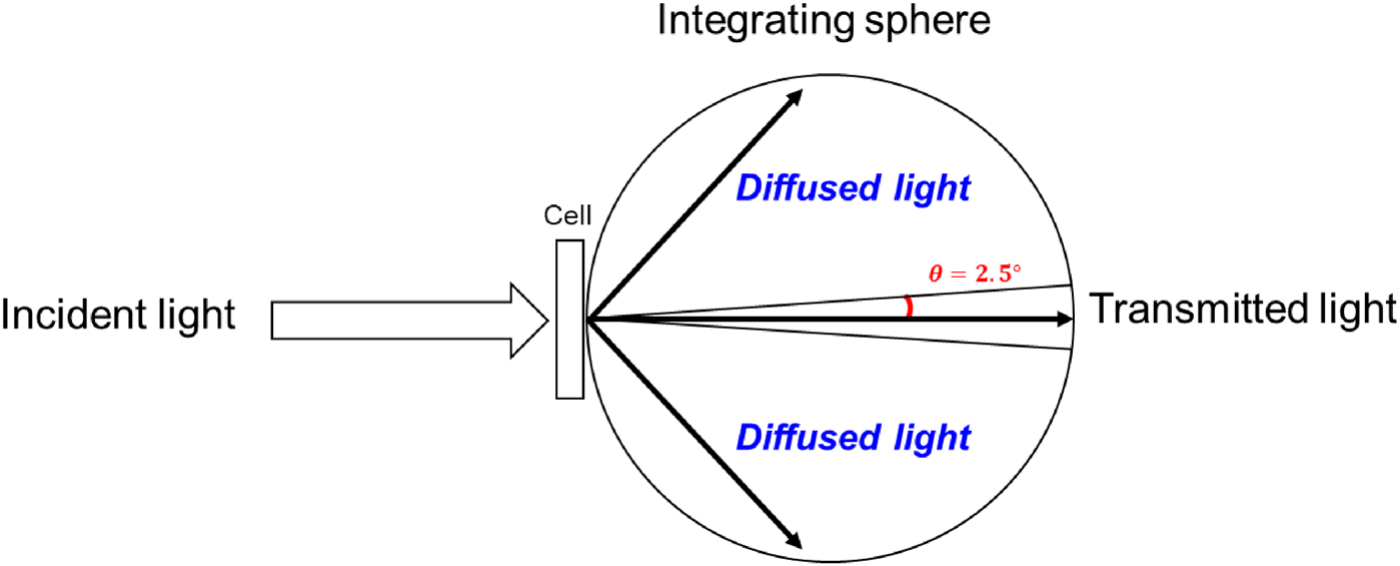
Calculating condition for the grating cells haze value.

The haze values of the LC grating cells were calculated to determine their haziness. To evaluate the optical performance, we introduced the total, specular, and diffuse transmittance and haze. Specular [diffuse] transmittance *T*_*s*_ [*T*_*d*_] refers to the ratio of the power of the beam that emerges from a sample cell, which is parallel (within a small range of angles of 2.5°) [not parallel] to a beam entering the cell, to the power carried by the beam entering the sample. The total transmittance *T*_*t*_ is the sum of the specular transmittance Ts and the diffuse transmittance *T*_*d*_. The haze *H* can be calculated as *H* = *T*_*d*_/*T*_*t*_. In our calculation, specular transmittance was calculated by integrating the intensity with a range of 2.5° as shown in Fig. 3. The *T*_*d*_ was calculated by the difference between *T*_*t*_ and the *T*_*s*_.

**Figure 4:**
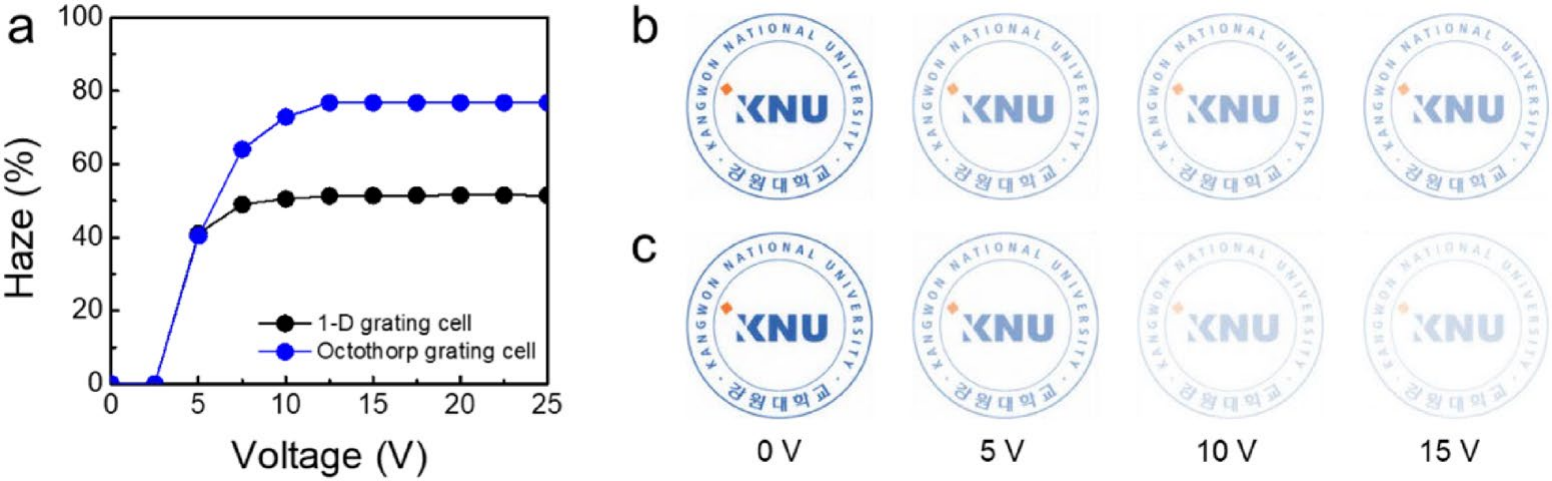
(**a**) Haze values of 1-D grating cell and octothorp grating cell. The calculated images of the (**b**) 1-D grating and (**c**) octothorp grating cells with the KNU logo.

At an applied voltage of 10 V, the 1-D grating cell had a haze of 51.2%, whereas the octothorp grating cell had a higher haze of 76.7% as shown in Fig. 4a. This is because the octothorp grating cell has a much larger spatial phase difference, independent of the azimuthal angle. The octothorp grating cells accounted for 25.5% higher haze values than the 1-D grating cell. This is comparable to LC smart windows based on light scattering, such as polymer-dispersed liquid crystal (PDLC) or polymer-networked liquid crystal (PNLC) cells, which have been previously reported. Because the proposed LC cell does not contain any polymer matrices, haze in the opaque state is predominantly caused by the electric-field-induced periodic continuous LC profile diffraction of the white incident light. As a result, when compared to other LC smart windows, the proposed cell offers benefits such as low angle dependence, high stability, low operating voltage, fast response time, and ease of fabrication. Using image analysis in TechWiz LCD 3D, we estimated the images of the LC grating cells placed on top of printed paper (KNU logo) at various applied voltages. When a voltage of 15 V was applied, both the grating cells became opaque. Figure 4b and c show the proposed grating cell was hazier than the 1-D grating cell.

**Figure 5:**
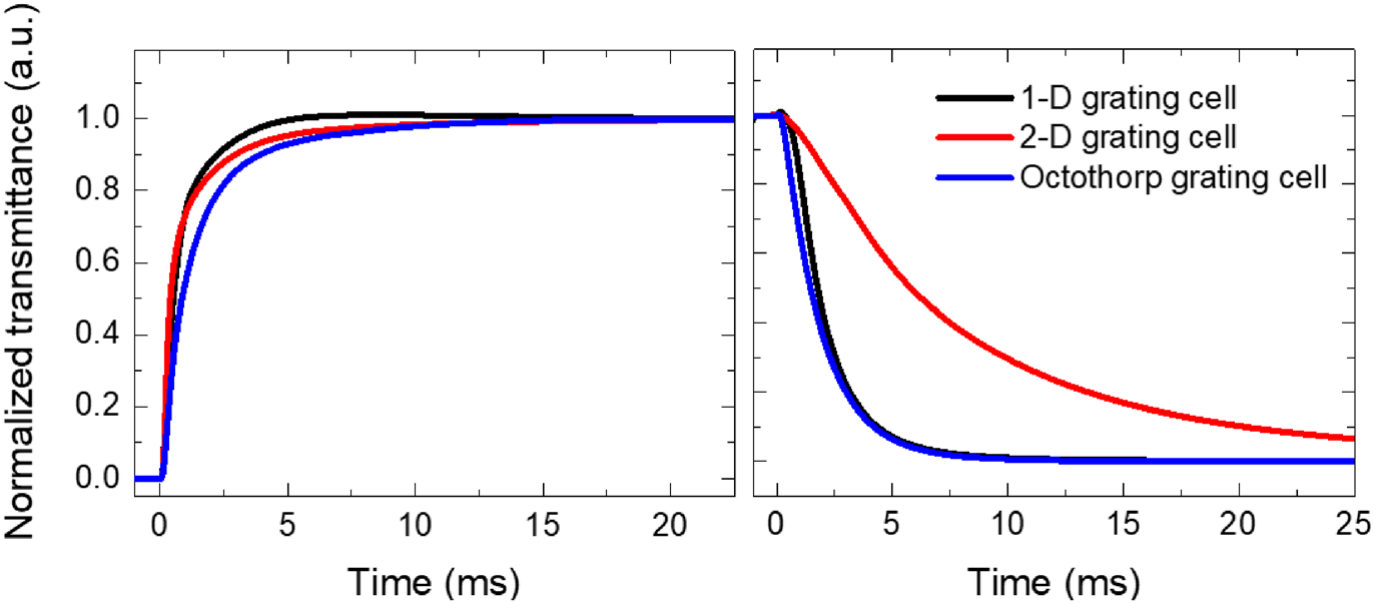
Calculated response time of LC grating cells (the 1D, the 2D, and the octothorp cells).

A fast response time is one of the most important requirements for window-display applications. The dynamic switching behavior of the proposed LC cell was investigated (Fig. 5). The proposed grating cell had a total response time of 7.57 ms, which is substantially faster than the existing LC smart windows, including cholesteric liquid crystal, polymer-network liquid crystal, and polymer-dispersed liquid crystal cells, which have response times of several hundred milliseconds^19,37,38^. In addition, the response times for the 1-D and 2-D grating cells were examined. The calculated turn-on [turn-off] time for 1-D, 2-D, and octothorp grating cells were 2.23 ms [3.56 ms], 3.23 ms [18.6 ms], and 3.79 ms [3.78 ms], respectively. The top and bottom patterned electrodes were used in the 2-D grating cell, with the top patterned electrode receiving voltage in the *x*-direction and the bottom patterned electrode receiving voltage in the *y*-direction. As a result, the LCs in the bulk region of the 2-D grating cell were formed in a random direction, whereas the suggested LC direction of the grating cell had an *x*- and *y*-direction owing to the single bottom patterned electrode.

**Figure 6:**
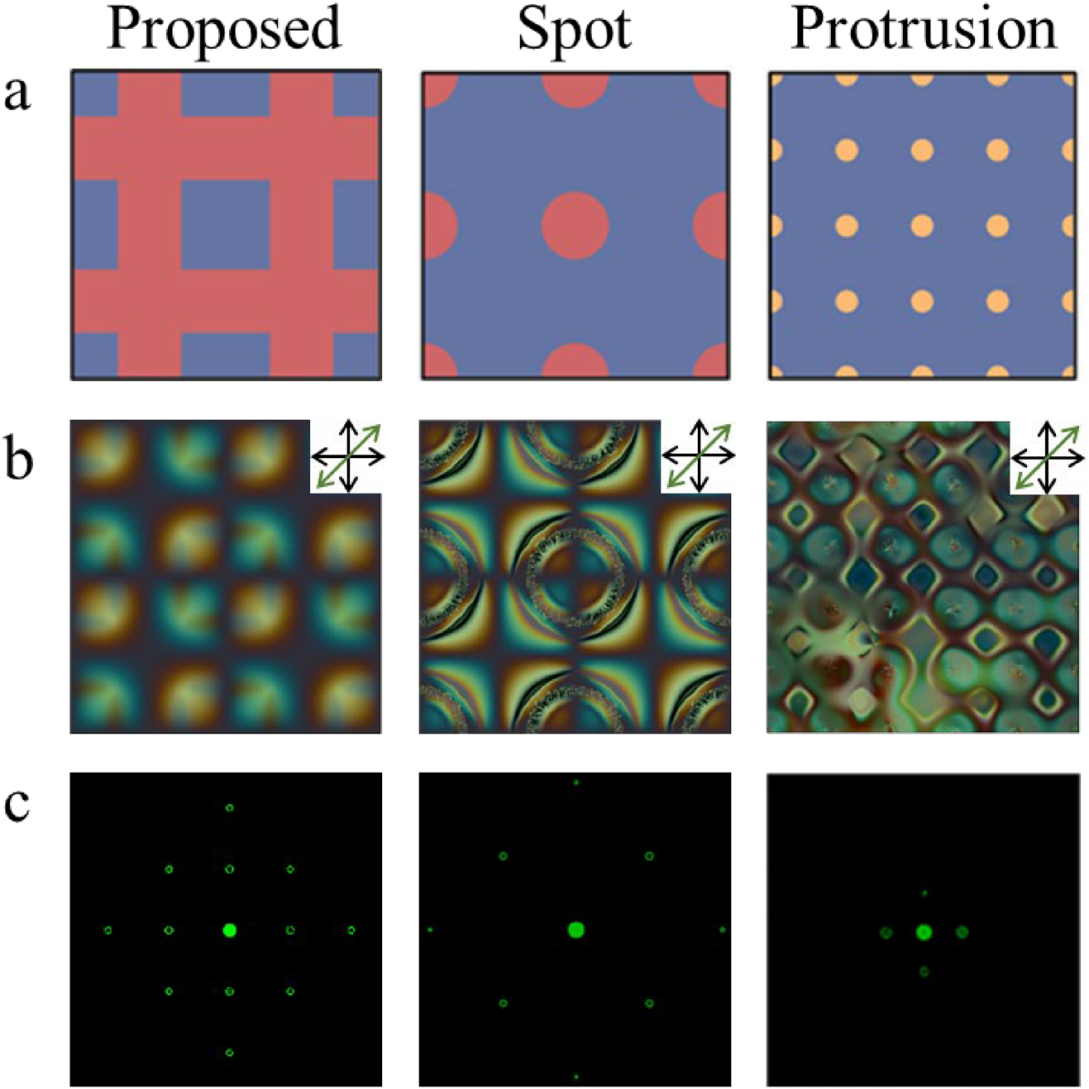
LC grating cells with octothorp and spot patterned electrode and protrusion grating cells without patterned electrode. (**a**) Schematic of the structures; (**b**) POM images; (**c**) Diffraction patterns at maximum haze value.

The proposed cell can make a 2-D phase grating effect by consisting of patterned electrodes in only one substrate. We have additionally demonstrated a few more devices that can produce the 2-D grating effect with structures in one substrate (the spot and protrusion grating cells). Figure 6a shows schematics of the LC grating cell with octothorp and spot-patterned electrodes and the protrusion grating cell without patterned electrodes. The red, blue, and yellow colors in Fig. 6a depict a patterned electrode, common electrode, and insulator, respectively. Compared to the proposed grating cell, the spot grating cell consisted of a circle-patterned electrode. The electrode in the spot grating cell was formed by swapping the common and patterned electrodes, unlike the proposed grating cell. The protrusion grating cell has the same spot structure. It should be noted that the protrusion grating cell does not use a patterned electrode.

Figure 6b shows POM images of crossed polarizers and a full-wave plate (45°) under the same conditions as in Fig. 2a. The POM image of the spot cell was slightly different from that of the octothorp cell owing to the formation of additional virtual walls. This difference results in the decrease of the effective period by half^33^. Therefore, the diffraction angle of the spot grating cell increases owing to the decrease in the effective period (Fig. 6c). In the protrusion grating cell, which does not use a patterned electrode, the direction of the electrode field is the same, regardless of the position. In addition, LCs near the protrusion form a pretilt angle, which can provide a direction to other LCs in the bulk region to create the 2-D effect as LCs have randomly lied along the direction^36,39^. By increasing the voltage in the protrusion grating cell, we can observe that the surrounding LCs form new domains by lying in a similar direction, as shown in Fig. 6b. It should be noted that the domain size can be changed with time and applied voltage, which can result in low reliability. In the protrusion grating cell, we reduced the period required to achieve a sufficient diffraction effect. We expected that the reduced period would result in a large diffraction angle; however, the diffraction angle was found to be reduced. Because the LC domains were not formed by the electric field from the patterned electrode, the bulk LCs followed the LCs near the protrusion, and the domains were broken and merged by sounding defects^36^. Therefore, it had a large domain size.

**Figure 7:**
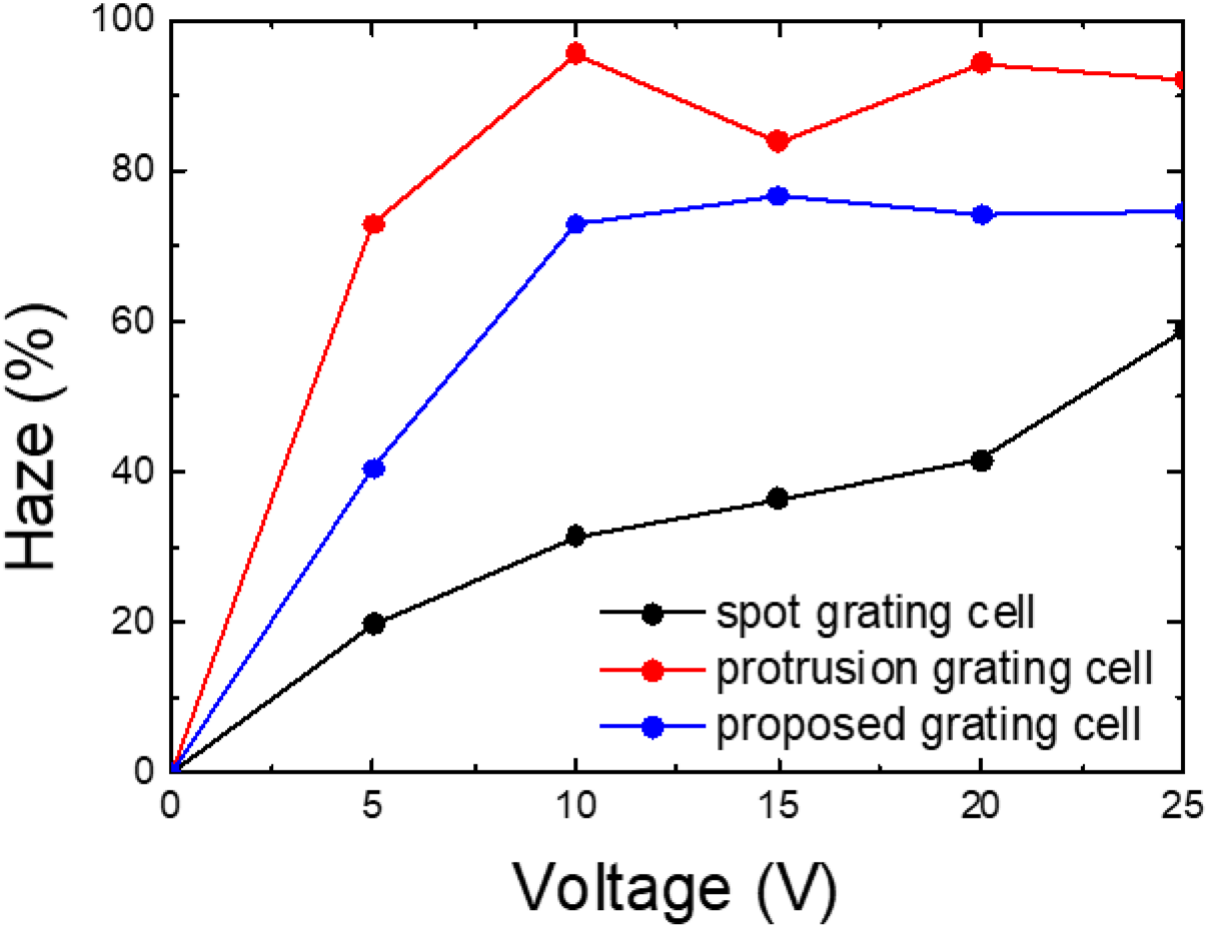
Calculated haze values of the spot, protrusion, and proposed grating cells as a function of the applied voltage.

We have calculated the haze value of the LC grating cells using the experimental setup as shown in Fig. 3. The maximum haze values of the proposed, spot, and protrusion grating cells were 76.7, 70.45, and 95.56% at 12.5, 35, and 10 V, respectively as shown in Fig. 7. The spot grating cell has a high operating voltage because the area, LCs switched by the elastic energy (blue region in Fig. 6a), is larger than the proposed cell. In addition, the calculated response time profiles of the spot and protrusion grating cells. The total response time is 474.178 and a hundred milliseconds, respectively. In the case of the spot and protrusion grating cell, response time is very slow response time. Because the spot grating cell has many bulk LCs by the circle patterned electrode, and the protrusion grating cell is switched LCs using insulator and rubbing angle without the patterned electrode.

## Conclusion

We demonstrate the electro-optical characteristics of a vertically aligned LC grating cell with an octothorp electrode for window display applications. The proposed grating cell shows a higher haze than that of the 1-D grating cell owing to the large spatial phase difference in any direction. Our proposed grating cell has the advantage of high fabricability because the proposed grating cell has crossed interdigitated electrodes only in the bottom substrate, easy driving, low power consumption, and fast response time than the 2-D grating cell (similar to 1-D grating cell). Therefore, we expect our proposed grating cell to have various applications, such as military devices, augmented reality, virtual reality devices, and window applications that require fast response.

## Data Availability

The datasets used and/or analysed during the current study available from the corresponding author on reasonable request.
